# Human Activity Recognition via Attention-Augmented TCN-BiGRU Fusion

**DOI:** 10.3390/s25185765

**Published:** 2025-09-16

**Authors:** Ji-Long He, Jian-Hong Wang, Chih-Min Lo, Zhaodi Jiang

**Affiliations:** 1 School of Computer Science and Technology, Shandong University of Technology, Zibo 255000, China; 23505020692@stumail.sdut.edu.cn (J.-L.H.); zdjiang1011@163.com (Z.J.); 2 Department of Digital Multimedia Design, School of Innovative Design and Management, National Taipei University of Business, No. 321, Sec. 1, Jinan Rd., Zhongzheng District, Taipei City 100025, Taiwan

**Keywords:** human activity recognition, temporal convolutional network, bidirectional gated recurrent unit, attention mechanism, sensor data sequences

## Abstract

With the widespread application of wearable sensors in health monitoring and human–computer interaction, deep learning-based human activity recognition (HAR) research faces challenges such as the effective extraction of multi-scale temporal features and the enhancement of robustness against noise in multi-source data. This study proposes the TGA-HAR (TCN-GRU-Attention-HAR) model. The TGA-HAR model integrates Temporal Convolutional Neural Networks and Recurrent Neural Networks by constructing a hierarchical feature abstraction architecture through cascading Temporal Convolutional Network (TCN) and Bidirectional Gated Recurrent Unit (BiGRU) layers for complex activity recognition. This study utilizes TCN layers with dilated convolution kernels to extract multi-order temporal features. This study utilizes BiGRU layers to capture bidirectional temporal contextual correlation information. To further optimize feature representation, the TGA-HAR model introduces residual connections to enhance the stability of gradient propagation and employs an adaptive weighted attention mechanism to strengthen feature representation. The experimental results of this study demonstrate that the model achieved test accuracies of 99.37% on the WISDM dataset, 95.36% on the USC-HAD dataset, and 96.96% on the PAMAP2 dataset. Furthermore, we conducted tests on datasets collected in real-world scenarios. This method provides a highly robust solution for complex human activity recognition tasks.

## 1. Introduction

With the rapid development of Internet of Things (IoT) technology and the proliferation of wearable devices, human activity recognition (HAR) using inertial data has become an important research direction in the field of artificial intelligence due to the low cost and portability of wearable devices [1]. Human activity recognition systems mainly include two types of methods, video-based and sensor-based, with the latter utilizing multi-inertial measurement unit (IMU) data fusion to handle the complexity of sensor data. Sensor-based methods demonstrate broad application prospects in fields such as healthcare monitoring (e.g., fall detection for elderly individuals, chronic disease management) [2], smart home control (automatic adjustment based on user behavior) [3], and sports science analysis (athlete posture optimization) [4]. However, constructing efficient and robust recognition models remains a major challenge in current research due to the characteristics of sensor data such as high dimensionality (multiple data channels), strong temporal dependency (rapidly changing data with strong sequential correlations), and susceptibility to noise interference.

Traditional HAR methods primarily rely on manual feature engineering combined with shallow machine learning models (e.g., Random Forests, Support Vector Machines). This approach requires domain experts to invest significant time in designing time-domain features (e.g., mean, variance), frequency domain features (e.g., Fourier coefficients), and time–frequency domain features (e.g., wavelet transforms). The performance of these methods is constrained by the comprehensiveness of feature design [5].

In recent years, deep learning techniques have achieved significant breakthroughs in HAR tasks through their end-to-end feature learning mechanism. Convolutional Neural Network (CNN) can effectively extract local spatial patterns from sensor signals via its local receptive fields, but struggles to capture long-term temporal dependency features [6]. Recurrent Neural Network (RNN) and its variants (LSTM/GRU) can capture temporal dynamics. However, traditional LSTM suffers from parameter redundancy due to its complex gating mechanisms. In contrast, the Gated Recurrent Unit (GRU) significantly reduces computational complexity by streamlining the gating structure (merging update and reset gates) while maintaining temporal feature extraction capabilities, making it more suitable for resource-constrained mobile scenarios [7].

The rise of Temporal Convolutional Networks (TCN) has introduced new research pathways in the field of time-series signal analysis. Based on the causal dilated convolution structure of TCN, the model can efficiently capture long-term dependencies while strictly maintaining temporal causality by progressively expanding the receptive field through exponentially increasing dilation rates [8,9,10]. However, as demands evolve toward modeling more complex long-term dynamic patterns, the single TCN architecture still has room for optimization in terms of feature interaction depth and time-varying state modeling.

Existing research often overlooks the varying importance of key time periods in sensor signals, failing to effectively integrate the local feature extraction strengths of CNN with the global temporal feature extraction advantages of Recurrent Neural Networks. Simultaneously, some studies directly use the output of RNN as final features for classification, lacking sufficient capability to suppress noisy signals and failing to effectively eliminate redundant components in the features [11].

Therefore, this study proposes a multi-level fusion model named TCN-GRU-Attention-HAR (TGA-HAR). The TGA-HAR model integrates Temporal Convolutional Network (TCN), Bidirectional GRU, and Attention mechanism. First, the dilated causal convolution structure of TCN expands the receptive field. By stacking residual connections, the model achieves multi-scale temporal feature extraction, overcoming the locality constraints of traditional CNN. Second, a Bidirectional GRU (BiGRU) layer is introduced to capture bidirectional contextual dependencies in sequential data. Its streamlined gating mechanism reduces the number of parameters while improving training efficiency. Finally, the Attention mechanism dynamically allocates weights to different time steps, amplifying key motion features and suppressing noise interference. This hierarchical architecture enables the model to simultaneously capture local morphological features and long-term temporal patterns in sensor data, forming complementary and enhanced feature representations.

This study adopts an end-to-end learning framework, directly inputting preprocessed time-domain data into the deep learning model. Through its multi-layer network structure, the model automatically extracts spatiotemporal features of activities and outputs classification results, eliminating the manual statistical feature design process in traditional methods. This approach fully leverages the representation learning capability of deep learning, thereby enhancing the model’s adaptability to complex motion patterns.

To validate the effectiveness and generalization capability of the model, this study systematically evaluates the proposed method on three public datasets (PAMAP2, USC-HAD, and WISDM). These datasets exhibit significant heterogeneity in terms of sensor types (Inertial Measurement Unit (IMU)/accelerometer/gyroscope), activity categories (basic daily activities/complex motions), and data collection environments (laboratory-controlled/daily-life settings). We used the Real-World IDLab Dataset to test the performance of the TGA-HAR model in real-world conditions. Through cross-dataset comparative experiments, this study not only verifies the model’s superior performance in single-scenario evaluations but also rigorously examines its robustness under different sensor configurations and varying behavioral complexity levels.

The main contributions of this study are summarized as follows:

- This study proposes a TCN-GRU-Attention multidimensional feature fusion framework, which realizes complex human activity recognition by hierarchically integrating local features extracted from sensor data with global temporal relational features.
- This study designs a collaborative optimization strategy that combines TCN-BiGRU and the Attention mechanism: TCN extracts multi-scale local features, BiGRU captures bidirectional global temporal dependencies, and the Attention mechanism dynamically assigns weights to key time steps, thereby enhancing the discriminative power of feature representations.
- This study systematically verifies the effectiveness of fusing multidimensional inertial data. Experiments on the WISDM, USC-HAD, PAMAP2, and Real-World IDLab Dataset, which cover activity categories from basic daily behaviors to high-intensity exercises, confirm the model’s superior performance in single scenarios and robust generalization across diverse sensor configurations and activity complexities.

The subsequent sections of this paper are organized as follows: Section 2 reviews the research progress of deep learning in the field of HAR; Section 3 elaborates on the model architecture and design of TCN-GRU-Attention; Section 4 introduces the experimental datasets, experimental design, and experimental results; Section 5 summarizes this paper.

## 2. Related Work

**Figure 1:**
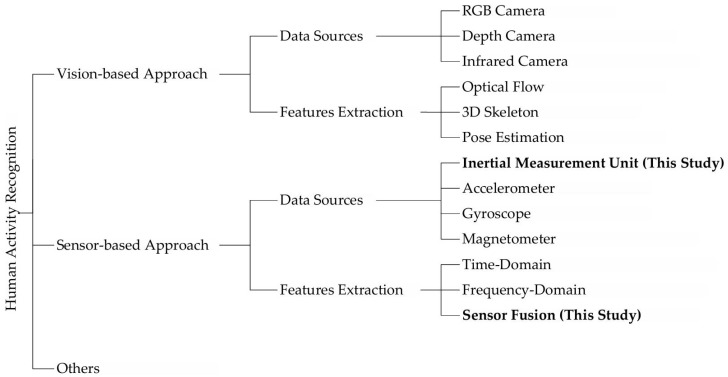
Comparative diagram of research on human activity recognition based on data sources and feature extraction.

Figure 1 presents a comparison of research on human activity recognition based on data sources and feature extraction methods. Human activity recognition primarily includes two approaches: vision-based methods and sensor-based methods. Vision-based methods utilize data sources such as RGB cameras [12], depth cameras [13], and infrared cameras [14], extracting features through optical flow [15], 3D skeletons [16], and pose estimation [17] to analyze human motions. Sensor-based methods rely on inertial measurement units (IMUs) [18], accelerometers [19], gyroscopes [20], and magnetometers [21] for data acquisition, employing feature extraction techniques such as time-domain analysis [22], frequency domain analysis [23], and sensor fusion [24] to achieve efficient human activity recognition. In recent years, activity recognition technology based on WiFi channel state information (CSI) has also attracted attention due to its non-intrusive nature and environmental universality. It achieves detection by analyzing changes in the multipath propagation of wireless signals under the interference of human movement [25,26,27].

However, vision-based methods suffer from limitations including high hardware costs (expensive high-definition cameras), poor privacy protection, and strong environmental dependencies (e.g., sensitivity to background lighting and susceptibility to occlusion). Activity recognition technology based on WiFi CSI has limitations of sensitivity to environmental noise, strong hardware dependence, and high computational complexity. Therefore, this study focuses on sensor-based methods for human activity recognition, specifically utilizing IMUs and multi-sensor fusion to accomplish complex human activity recognition.

Table 1 presents several existing research approaches in human activity recognition (HAR), their used datasets, data sources, and advantages. The Multi-branch CNN-BiLSTM achieved significant progress in multidimensional data fusion strategies for HAR based on inertial sensor data. This hybrid architecture combining multi-branch convolutional neural networks (CNN) with bidirectional long short-term memory networks (BiLSTM) could extract multi-scale temporal features using convolutional kernels of different sizes [28]. The Attention-based Multi-head CNN incorporated a squeeze-and-excitation attention module to dynamically adjust sensor channel weights, effectively suppressing noise interference [29]. The CNN-LSTM-Self-Attention model extracted joint temporal features from triaxial acceleration, gyroscope, and linear acceleration signals. This hybrid model achieved favorable recognition performance on the self-collected H-Activity dataset, demonstrating the effectiveness of multimodal data fusion for complex action classification [30].

To address feature space optimization, the spatial attention CNN + genetic algorithm employed a spatiotemporal encoding method based on continuous wavelet transform to convert sensor signals into 2D images, combined with a spatial attention mechanism. This approach integrated genetic algorithms for optimal feature subset selection [31]. CNN-BiGRU utilized a cascaded model combining Bidirectional Gated Recurrent Units (BiGRU) with convolutional neural networks (CNN). It enhanced long-range feature memory through bidirectional temporal dependency extraction [32]. TCN-Attention-HAR integrated a multi-scale Temporal Convolutional Network with a self-attention mechanism, employing knowledge distillation to compress model parameters. This demonstrated synergistic advantages in temporal feature extraction and feature compression [33].

For global dependencies, the Temporal-Channel Dual-Branch Self-Attention Network independently processed temporal dimensions and cross-sensor channel interactions using dual-branch convolutional self-attention networks. This architecture achieved higher average recognition accuracy than traditional LSTM and Transformer models on seven public datasets including MHEALTH [34]. Transformer applied self-attention mechanisms to analyze accelerometer and gyroscope signals, successfully capturing global cross-timestep dependencies. It achieved high recognition accuracy on the KU-HAR dataset, providing a novel paradigm for end-to-end multi-sensor fusion [35].

## 3. Methods

**Figure 2:**
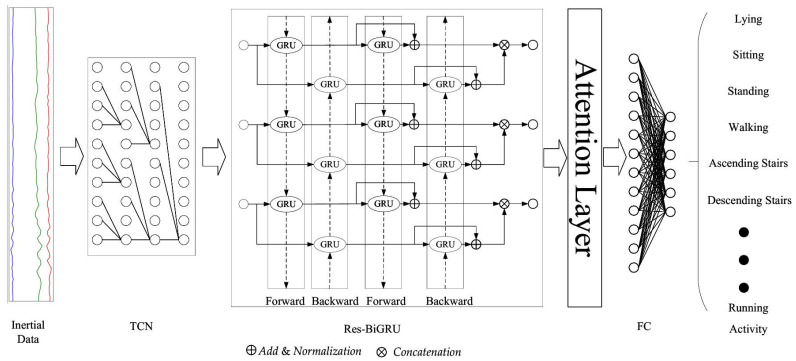
TGA-HAR Model.

This study integrates TCN, BiGRU, and the attention mechanism to propose the TGA-HAR model. Through hierarchical fusion of TCN, BiGRU, and the attention mechanism, a three-stage feature extraction and dynamic weighting framework is constructed to achieve recognition of complex human activities. The overall architecture is shown in Figure 2.

In the proposed TGA-HAR model, raw data acquired from sensors are segmented via time windows to ensure each data segment contains complete time-series information. The colored lines in the figure correspond to data collected from different channels, respectively. Subsequently, the time-window-segmented data is fed into the TCN module. Leveraging its characteristic dilated causal convolution, the TCN module extracts multi-scale temporal features. After feature extraction, the processed data is input into the residual BiGRU module to further capture long-term dependencies and forward-backward temporal relationships within the data. The Res-BiGRU output is then delivered to the attention layer. Through learnable fully connected operations, the attention layer achieves adaptive dynamic weight allocation, enhancing the model’s focus on critical features. Finally, the weighted data undergoes dimensionality reduction via a fully connected layer to accomplish effective human activity recognition.

Table 2 presents symbols used in Section 3 and their corresponding descriptions.

### 3.1. Temporal Convolutional Network

**Figure 3:**
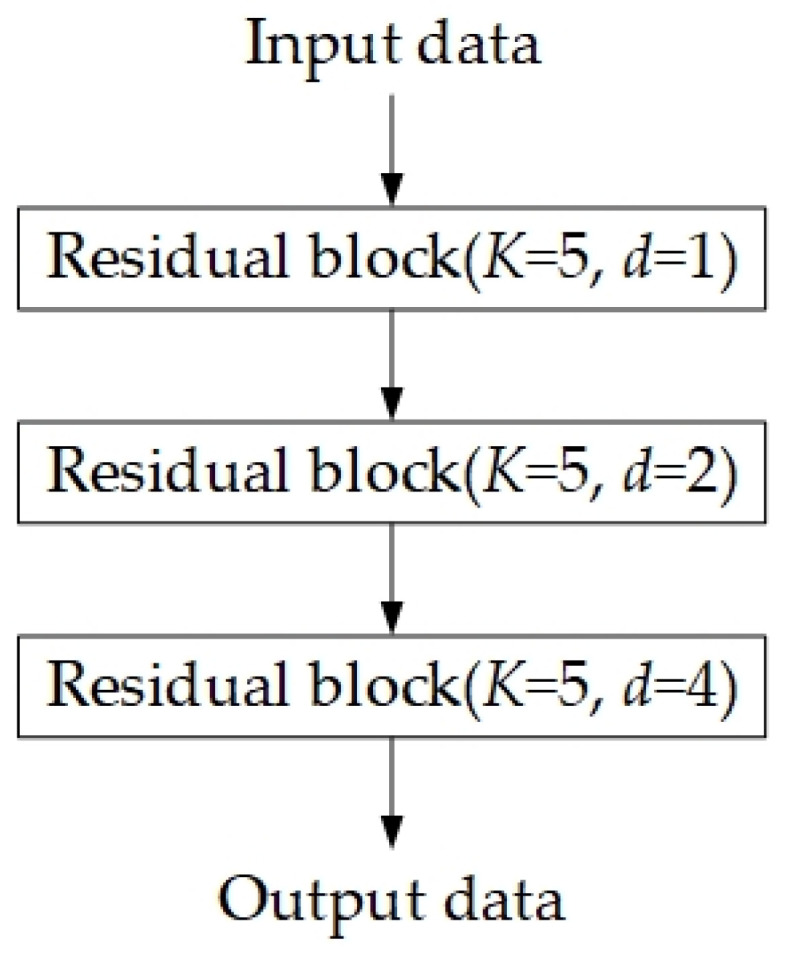
Architecture of the TCN model.

Temporal Convolutional Network (TCN) addresses the fundamental limitations of traditional convolutional neural networks in extracting features from time-series data, particularly their inability to capture long-term dependencies [36,37]. The fixed kernel size of traditional CNNs restricts their capacity to learn long-range temporal patterns, while TCN overcomes this issue by integrating causal dilated convolution and residual connections. TCN constructs a convolutional architecture specifically designed for sequence data. The TCN framework is illustrated in Figure 3 [36]. It ensures identical lengths for input and output sequences while strictly adhering to causality—where outputs depend solely on current and historical inputs—thereby preventing future information leakage. Here, K denotes the convolution kernel size, and d represents the dilation factor.

#### 3.1.1. Causal Dilated Convolution

Causal convolution is the core design that distinguishes Temporal Convolutional Networks (TCN) from traditional convolutional methods, ensuring strict causality in temporal feature extraction. This mechanism constrains the orientation of the convolutional kernel’s receptive field, enabling the model to utilize only current and historical information for prediction, thereby preventing future data leakage from affecting time series.

**Figure 4:**
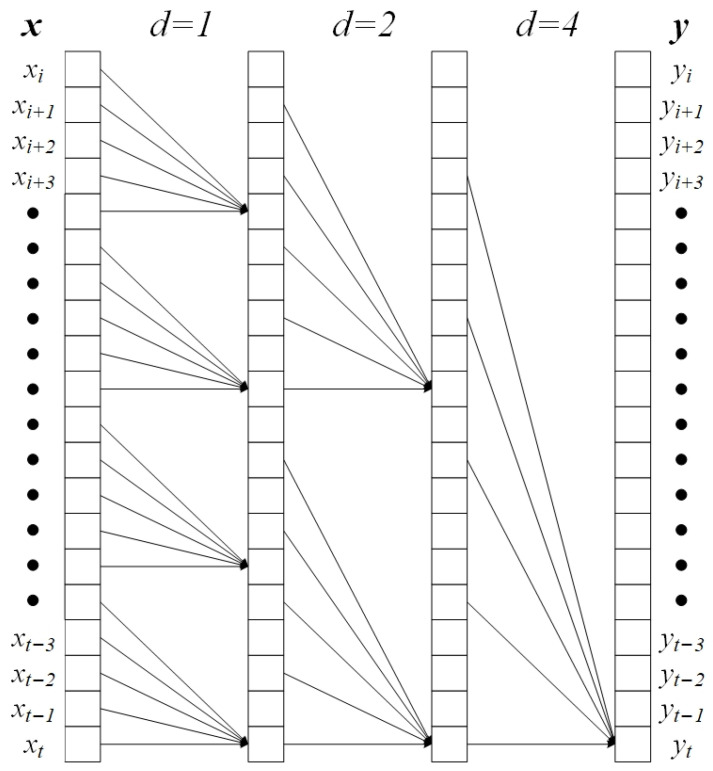
Causal dilated convolution (K = 5).

Dilated convolution (also known as atrous convolution) constitutes an essential component of TCN. By introducing a dilation factor d, it expands the receptive field of the convolutional kernel to capture long-range temporal dependencies without increasing the number of parameters, as illustrated in Figure 4 [37].

The causal dilated convolution operation is expressed by Equation (1) [36].

(1) $$y_{t}^{\left(n\right)}=\sum_{i=0}^{K-1}\;w_{i}\;x_{t-d\times i}^{\left(n\right)}\;,\;\;\;\;\;\;\;\;\;K=5,\;d\in \left\{1,\;2,\;4\right\},n\in \{1,\;2,\;3\}$$

Here, d denotes the dilation factor, set to {1, 2, 4} in this study to control the spacing between convolutional kernel elements. The setting of this dilation factor was selected with reference to [36]. Dilation factors configured as 1, 2, and 4 can effectively facilitate TCN in extracting multi-level local information, thereby aiding the TGA-HAR model in achieving improved performance. $x_{t-d\times i}^{\left(n\right)}$ represents the input data at time point (t−d×i) for the n-th convolutional layer. When t−d×i<0, a zero-padding strategy is employed to complete the computation. $w_{i}$ indicates the convolutional kernel weight at position i. The output of causal dilated convolution, $y_{t}^{\left(n\right)}$ depends entirely on inputs at time step t and earlier, strictly preventing any introduction of future temporal data. Simultaneously, by exponentially increasing the dilation factor between layers, the receptive field of TCN expands exponentially with network depth. This significantly enhances the model’s capability to capture long-term temporal patterns.

#### 3.1.2. Activation Function

Within the TCN architecture, we employ a modified Swish activation function, whose mathematical definition is given by Equation (2) [38].

(2) $$v^{\left(n\right)}=y^{\left(n\right)}\cdot sigmoid\left(\beta \;y^{\left(n\right)}\right)=y^{\left(n\right)}\cdot \frac{1}{1+e^{-\left(\beta \;y^{\left(n\right)}\right)}},\;\;\;\;\;\;\;\;\beta =1,\;\;\;\;\;\;n\in \{1,\;2,\;3\}$$

Here, we set β=1. This equation represents a specific instance of the Swish function when *β* = 1, achieving dynamic nonlinear feature transformation through element-wise multiplication of input values with their Sigmoid-gated weights. Compared to conventional activation functions like ReLU, it retains the core advantages of Swish while simplifying parameter configuration.

#### 3.1.3. Residual Connections

Residual connections address the issues of vanishing gradients and network performance degradation by propagating shallow-layer features directly to deeper layers via skip connections, whose mathematical formulation is defined by Equation (3) [39].

(3) $$v^{\left(n+1\right)}=F\left(v^{\left(n\right)},\left\{\omega \right\}\right)+\omega^{\prime }\;v^{\left(n\right)},\;\;\;\;\;\;n\in \{1,\;2,\;3\}$$

Here, $v^{\left(n\right)}$ denotes the input data to the residual block. $v^{\left(n+1\right)}$ represents the output data of the residual block, which serves as the input to the next residual block or the subsequent Res-BiGRU module. F() signifies the residual function composed of dilated causal convolution, batch normalization, and an activation function. {ω} corresponds to the convolutional kernel weight matrices, while $\omega^{\prime }$ is a linear projection matrix activated for dimensionality matching when input and output dimensions differ. In standard TCN implementations, $\omega^{\prime }$ is typically simplified to an identity matrix, as shown in Equation (4).

(4) $$v^{\left(n+1\right)}=F\left(v^{\left(n\right)},\left\{\omega \right\}\right)+v^{\left(n\right)},\;\;\;\;\;\;n\in \{1,\;2,\;3\}$$

**Figure 5:**
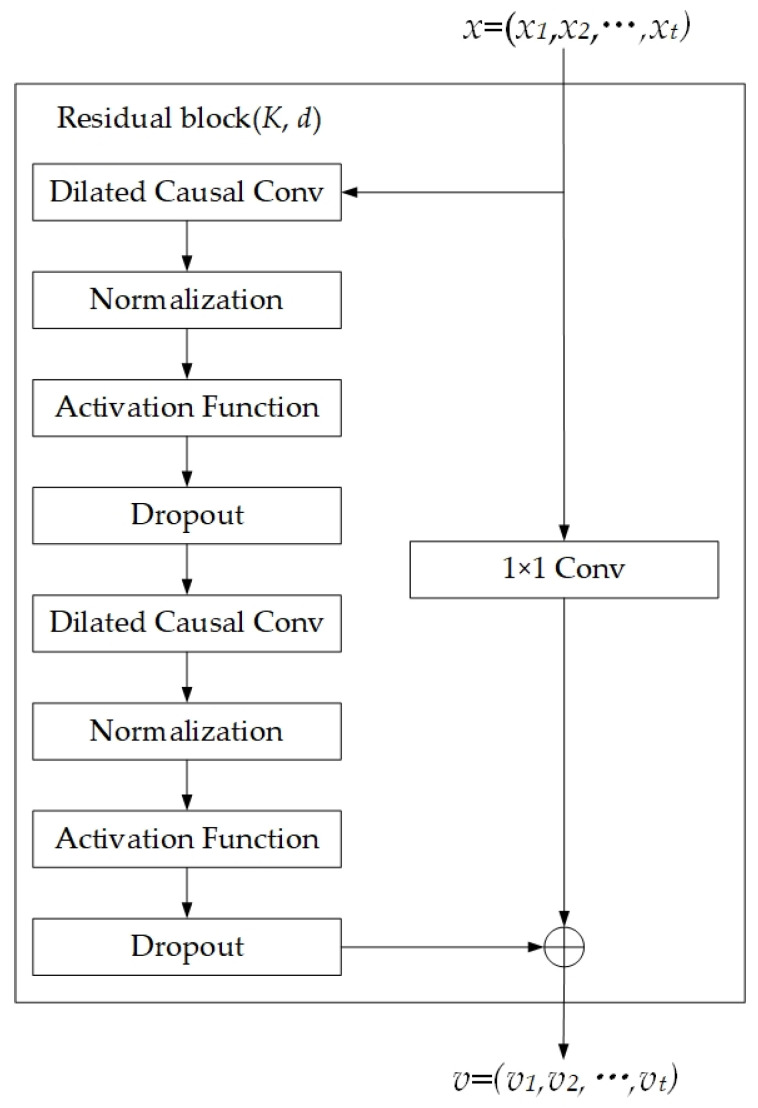
The residual block in TCN.

In the TCN architecture, the structure of the residual block is illustrated in Figure 5 [36]. The main path sequentially processes data through causal dilated convolution, normalization, activation function, and dropout, while the skip connection performs dimensionality transformation when necessary. Here, K denotes the convolution kernel size, and d represents the dilation factor. Here, we use a 1 × 1 convolution kernel to match the dimensions. The data processed by the causal dilated convolution is summed with the data processed by the 1 × 1 convolution kernel to realize the residual connection.

### 3.2. Bidirectional Gated Recurrent Unit

Considering the inherent bidirectional temporal relationships in human activities, after extracting temporal features via the TCN module, the TGA-HAR model employs a Bidirectional Gated Recurrent Unit (BiGRU) to capture bidirectional contextual dependencies in sequential data [40]. The BiGRU module strikes a balance between feature fusion efficiency and gradient stability.

#### 3.2.1. Gated Recurrent Unit

The Gated Recurrent Unit (GRU), proposed by Cho et al., is a variant of RNN. GRU was designed to address the vanishing gradient problem in traditional RNNs while simplifying the structure of LSTM networks [40]. Its core design utilizes two gating mechanisms—the update gate and reset gate—to dynamically regulate information flow, thereby effectively capturing long-term dependencies in sequential data. Compared with LSTM, GRU reduces the number of parameters by merging gating units, improves training efficiency, and achieves comparable performance in most tasks. The computational procedure of GRU is formalized in Equations (5)–(8) [41].

(5) $$z_{t}=\sigma (W_{z}\cdot [h_{t-1},v_{t}])$$

(6) $$r_{t}=\sigma (W_{r}\cdot [h_{t-1},v_{t}])$$

(7) $${\tilde{h}}_{t}=tanh(W\cdot [r_{t}⊙h_{t-1},v_{t}])$$

(8) $$h_{t}=(1-z_{t})⊙h_{t-1}+z_{t}⊙{\tilde{h}}_{t}$$

Here, $v_{t}\;$ denotes the current input. $z_{t}$ denotes the update gate state. $r_{t}$ denotes the reset gate state. ${\tilde{h}}_{t}$ denotes the candidate hidden state. σ() is the Sigmoid activation function. $W_{z}$, $W_{r}$, W denote the trainable weight matrices for the update gate, reset gate, and candidate hidden state, respectively. $h_{t-1}$ denotes the hidden state at the previous time step t−1. ⊙ denotes elementwise multiplication, and $h_{t}$ denotes the final state update.

#### 3.2.2. Residual Bidirectional Gated Recurrent Unit

**Figure 6:**
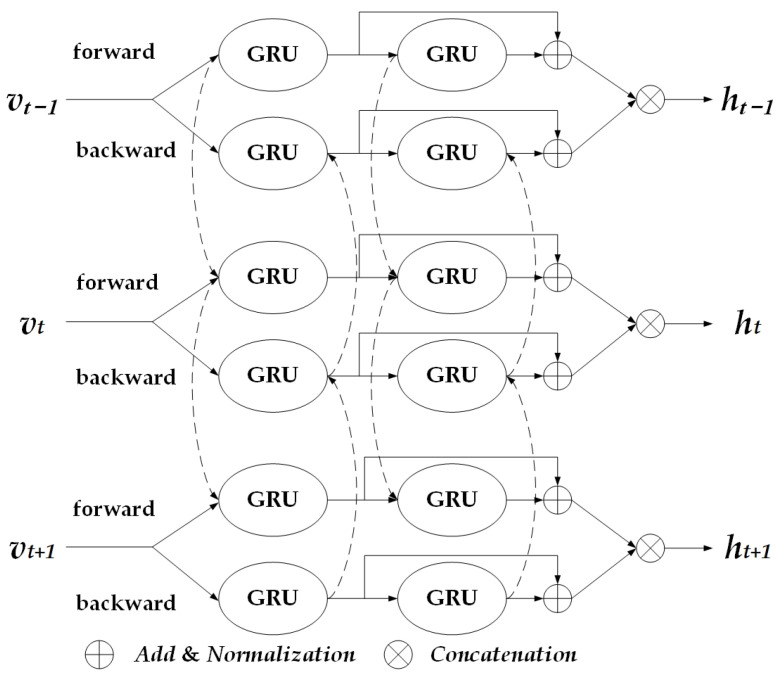
Res-BiGRU module.

For long-term feature extraction tasks, the Residual Bidirectional Gated Recurrent Unit (Res-BiGRU) architecture constructs an efficient feature learning system through hierarchically stacked bidirectional gating structures and layer-normalized residual connections, as illustrated in Figure 6 [40]. In this paper, the input dimension of Res-BiGRU is set to the number of channels of the convolution kernels in the TCN module.

The model employs Bidirectional Gated Recurrent Units (BiGRU) to perform parallel forward and reverse temporal scans. Leveraging gating mechanisms, it dynamically filters critical temporal features while deeply integrating bidirectional contextual dependencies of sequential data. To address gradient decay in deep networks, cross-layer residual paths embedded with normalization are designed. Low-level features, after undergoing the normalization operation, are summed and fused with the normalized output of the high-level network. This not only enhances the stability of gradient propagation but also facilitates the compatible interaction of cross-multiscale features. The stacked BiGRU modules achieve progressive abstraction of temporal representations. Gating units maintain efficiency in extracting long-range dependency features with streamlined parameters, while normalized residual connections enable nonlinear synergistic enhancement between local fine-grained patterns and global semantic structures through feature calibration.

This architecture combines bidirectional context awareness with normalized residual learning, forming a robust and efficient solution for temporal feature extraction.

### 3.3. Adaptively Weighted Attention Layer

To enhance the model’s focus on critical temporal features, we employ a dynamically weighted attention mechanism. This mechanism recalibrates the temporal characteristics of Res-BiGRU output features through learnable weighting parameters. Models utilizing attention mechanisms can simultaneously capture positional relationships between information elements and quantify the importance of distinct features. This study adopts a feedforward-structured dynamic attention mechanism, whose core concept leverages a learnable function to generate probabilistic weights for historical states, thereby achieving adaptive aggregation of contextual information.

The formulae for this mechanism are defined by Equations (9)–(11) [42].

(9) $$m_{t}=f(h_{t})$$

(10) $$\alpha_{t}=\frac{exp(m_{t})}{\sum_{i=1}^{T}\;exp(m_{i})}$$

(11) $$C=\sum_{t=1}^{T}\;\alpha_{t}m_{t}$$

Here, the function f( ) is implemented using a learnable fully connected layer, $h_{t}$ denotes the hidden state at time t output by Res-BiGRU. T denotes the number of hidden units in Res-BiGRU, set to 128 in this study. f( ) is learned through backpropagation.

In this paper, the feature $h_{t}$ with a dimension of [batch_size, seq_len, 128] is mapped to 1-dimensional attention scores $m_{t}$ via the fully connected layer f( ). The weight matrix of this fully connected layer has a dimension of [128, 1], and $m_{t}$ corresponds to a dimension of [batch_size, seq_len, 1]. Subsequently, weight normalization is performed, and a softmax operation is applied to $m_{t}$ along the “seq_len” dimension to obtain attention weights $\alpha_{t}$ (dimension: [batch_size, seq_len, 1]), which ensures the sum of the weights equals 1. Finally, weighted aggregation is implemented: after element-wise multiplication of $\alpha_{t}$ and $m_{t}$, summation is conducted along the seq_len dimension, yielding the final attention feature C (dimension: [batch_size, 128]). This feature C is then used for subsequent fully connected classification.

The attention weights α dynamically evolve during training. As the number of training epochs increases, the feature information C gradually becomes more discriminative and representative.

### 3.4. Classifier

In the model architecture of this study, the fully connected (FC) layer serves as the core component of the classifier, undertaking dual functions of feature mapping and classification decision-making. This module consists of three linear transformation layers that progressively compress the dimensionality of input features, ultimately mapping them to an output dimension matching the number of target activity categories. Each linear layer is followed by a LeakyReLU activation function to enhance nonlinear representational capacity. The mathematical formulation of LeakyReLU is defined by Equation (12) [43].

(12) $$\mathrm{LeakyReLU}\left(\zeta \right)=\left\{\begin{array}{c} \zeta ,\;\;\;\;\zeta \geq 0\\ \lambda \zeta ,\;\;\;\;\zeta <0 \end{array}\right.\;\;,\;\lambda =0.01$$

Here, the negative slope coefficient λ is set to 0.01. Compared to standard ReLU, LeakyReLU preserves minimal gradients for negative inputs (λ≪1), effectively mitigating the “neuron death” problem while maintaining sparse activation properties to suppress overfitting. This design significantly enhances the stability of gradient backpropagation in deep networks, particularly suitable for asymmetrically distributed data prevalent in temporal features.

During feature processing, the TCN module extracts local temporal patterns through dilated causal convolution. The Res-BiGRU module captures bidirectional long-term dependencies. The attention layer dynamically focuses on critical time steps.

The fully connected layer systematically integrates these high-level features. Its first two layers achieve dimensionality reduction and implement nonlinear transformations via LeakyReLU activation functions. This layered design reduces model parameters while preserving discriminative feature information, thereby enabling end-to-end classification.

## 4. Results

### 4.1. Dataset Description

The selection of public datasets for Human Activity Recognition (HAR) research requires balancing activity diversity, sensor modalities, and experimental reproducibility. This study employs three public datasets—WISDM, USC-HAD, and PAMAP2—representing low-cost single-sensor scenarios, laboratory-grade multimodal scenarios, and complex multimodal activity scenarios, respectively. Their complementary characteristics cover recognition requirements from basic actions to high-complexity activities, providing benchmark support for research on algorithm robustness and multimodal fusion.

The WISDM dataset was collected in a laboratory-controlled environment to ensure data quality. Acceleration data for six basic daily activities (walking, jogging, ascending stairs, descending stairs, sitting, standing) were gathered via smartphone accelerometers placed in the front pants leg pockets of 36 participants. With a sampling frequency of 20 Hz, the dataset comprises 1,098,209 samples (approximately 15.25 h of acceleration data). Data collection utilized only triaxial accelerometers, reflecting the typical configuration of early mobile devices [44,45]. Dataset specifications are summarized in Table 3.

USC-HAD (University of Southern California Human Activity Dataset) is a multimodal sensor dataset specifically designed for HAR, providing high-precision, diverse behavioral benchmarks for smart device interaction, healthcare monitoring, and robotic motion analysis. The dataset comprises data from 14 participants performing daily activities in a laboratory setting. Sensor data was captured by a 6-axis Inertial Measurement Unit (IMU)—including a triaxial accelerometer and gyroscope—fixed on the right front hip at a sampling frequency of 100 Hz. Each activity was repeated five times to enhance data diversity [46,47]. The average data collection duration per participant was approximately 6 h. Dataset specifications are detailed in Table 4, while the executed activities are listed in Table 5.

The PAMAP2 dataset targets complex activity recognition, capturing 18 activities in daily life environments using three Inertial Measurement Units (IMUs) (sampling frequency: 100 Hz) positioned on the wrist, chest, and ankle, and a heart rate sensor (sampling frequency: 9 Hz) worn by nine participants. The data collection duration per participant exceeded 10 h. Its multimodal signals—including acceleration, gyroscope, magnetometer, and heart rate data—support sensor fusion research but require handling high-frequency data and partial missing values. Consequently, it is exceptionally suitable for complex-scenario activity recognition and cross-modal analysis [48,49]. The dataset comprises 3,850,491 raw samples, with 2,724,817 valid activity data points (after preprocessing to remove invalid segments). Specifications are detailed in Table 6, and executed activities are listed in Table 7.

### 4.2. Data Preprocessing

**Figure 7:**
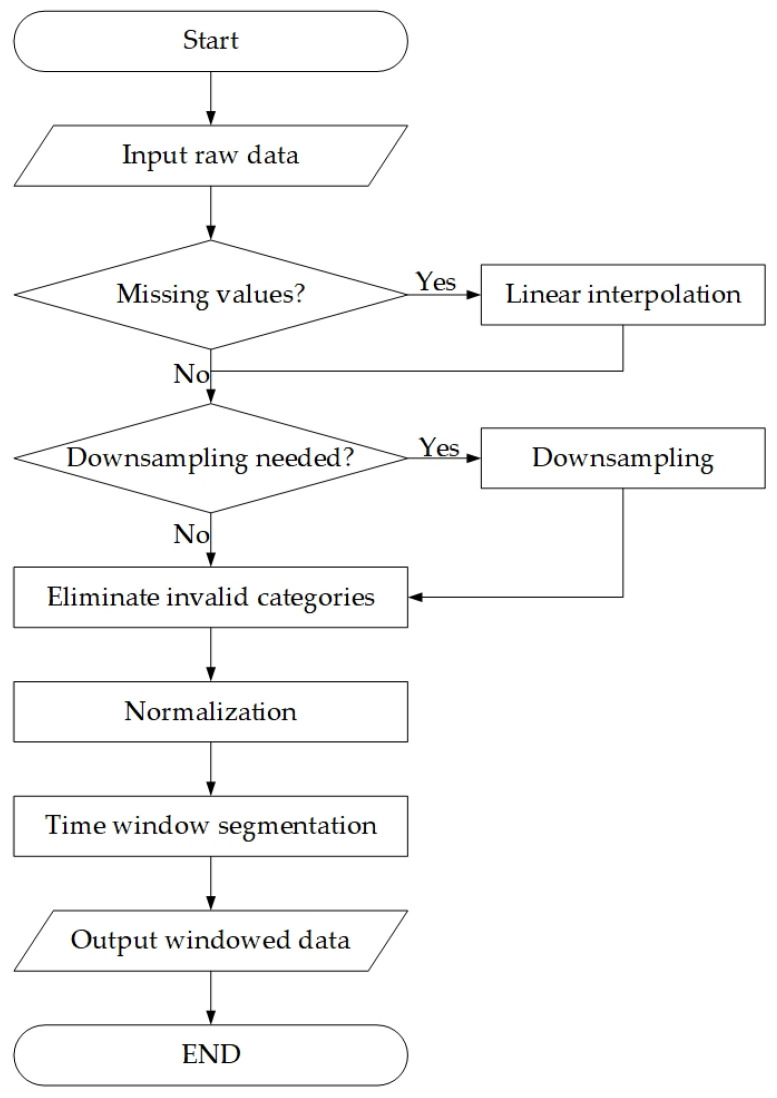
Dataset preprocessing workflow diagram.

To achieve human activity recognition, time-series signals collected from diverse sensor modalities are segmented into continuous samples via the sliding window technique. These samples are then normalized to [−1, 1]. The dataset preprocessing workflow is illustrated in Figure 7.

For the WISDM dataset, considering its low sampling frequency, a longer 4 s window (window length = 80) with 50% overlap rate is adopted during time window segmentation. This dataset approximates real-life scenarios, but its activity categories primarily focus on fundamental motion patterns. We split the training set and test set in an 8:2 ratio based on users. Specifically, the finally selected users for the test set are {1, 9, 11, 16, 19, 23, 28, 31}, and the data of the remaining users is used as the training set.

For the USC-HAD dataset, linear interpolation is first applied to repair missing values in sensor data, followed by downsampling to 50 Hz to balance computational efficiency and motion detail preservation. Time window segmentation employs a sliding window with a temporal stride of 128 (2.56 s) and 50% overlap rate, generating three-dimensional time-series data (samples × 128 × 6). We split the training set and test set in an 8:2 ratio based on users. Specifically, the finally selected users for the test set are {1, 10, 12}, and the data of the remaining users is used as the training set.

For the PAMAP2 dataset, we select 12 types of actions for classification, where the action indices correspond to those in Table 7: {1, 2, 3, 4, 5, 6, 7, 12, 13, 16, 17, 24}. We utilize three IMUs to extract raw triaxial acceleration (±16 G range) and angular velocity. Missing values are filled via linear interpolation, and valid activity segments are selected. We extract 18-dimensional time-series data from hand-, waist-, and ankle-mounted IMUs, using activity labels as supervision signals. To reduce computational complexity, the data is downsampled to 33 Hz and subsequently normalized to the range of [−1, 1]. During time window segmentation, a window length of 169 samples (corresponding to 5.12 s) is adopted, with a stride of 84 samples (resulting in a 50% overlap rate). We select the data of Subject 105 as the test set, while the data of the remaining subjects is used as the training set.

This preprocessing preserves the temporal dynamics of sensor time-series while enhancing data regularity through downsampling, normalization, and windowing. The preprocessed data provides high-quality input for subsequent end-to-end model learning. The sample counts of training and test sets for each preprocessed dataset are summarized in Table 8.

This study aims to further address issues commonly encountered in sensor time-series data in Human Activity Recognition (HAR) tasks, such as insufficient sample diversity and limited model generalization ability. To this end, we only perform data augmentation on the preprocessed training set data of three datasets: WISDM, USC-HAD, and PAMAP2. Specifically, random Gaussian noise is added, with a mean of 0 and standard deviation of 0.01. When combined with the downsampling, normalization, and sliding window preprocessing mentioned earlier, this data augmentation strategy can further improve the quality of input data, help the model better accommodate data variations in real-world scenarios, and provide more reliable support for subsequent end-to-end model learning.

### 4.3. Experimental Environment

The experiments in this study were conducted on a computational platform equipped with an NVIDIA GeForce RTX 4050 Laptop GPU and an Intel Core i7-13700H CPU. This computational unit accelerates matrix operations through the CUDA 11.8 parallel computing architecture. At the software level, the model was implemented using the PyTorch 2.2.0 deep learning framework, which is specifically optimized for mixed-precision training and dynamic computation graphs. PyTorch 2.2.0 is fully compatible with Python 3.11. The experimental environment configuration is detailed in Table 9.

The hyperparameter configuration for this experiment is detailed in Table 10. Parameter updates are performed using the Adam optimizer, with 400 training epochs and a fixed batch size of 300. The learning rate is set to 0.001, and the cross-entropy loss function is selected to optimize probability distribution discrepancies in classification tasks. This configuration balances training efficiency and convergence stability, empirically ensuring optimal model performance within reasonable computational overhead.

Table 11 details the holistic architectural configuration of the model. The backbone comprises a hierarchical feature processor integrating TCN, Res-BiGRU, and attention mechanism.

Input data first traverses a dilated convolutional stack with three residual TCN blocks. Each block employs 1D causal convolutions with 64 channels, kernel size = 5, and dilation factors sequentially set to 1, 2, and 4. The modified Swish activation function replaces conventional ReLU to enhance nonlinear representational capacity, supplemented with inter-layer batch normalization and regularization strategies.

TCN output features are fed into a skip-connected bidirectional GRU network. Feature data passes through the first BiGRU layer, whose outputs serve as inputs to the second BiGRU layer. The outputs of both layers undergo residual summation, forming a gradient-stable context-aware temporal feature extraction module.

In the attention layer, adaptively weighted attention normalizes and computes importance weights per timestep, enabling dynamic feature-weighted fusion. The classifier adopts a three-stage dimensionality reduction framework, with each fully connected layer followed by LeakyReLU activation (negative slope: 0.01) to balance information flow.

### 4.4. Evaluation Metrics

For classification tasks, the metrics used to evaluate model performance include accuracy, precision, recall, and F1 score.

Accuracy measures the proportion of correctly classified instances relative to the total population, with higher values indicating superior overall model performance. Precision quantifies the ratio of true positives to all predicted positives for a given class, reflecting its classification exactness. Recall represents the proportion of true positives correctly identified relative to all actual positives in that class, indicating coverage completeness. F1 score, defined as the harmonic mean of precision and recall, evaluates balanced classification performance per target class.

The mathematical definitions of these metrics are given in Equations (13)–(16) [50].

(13) $$Accuracy=\frac{\sum_{k=1}^{K}\;{TP}_{k}}{N}$$

(14) $$Precision=\sum_{k=1}^{K}\;\left(\frac{N_{k}}{N}\times \frac{{TP}_{k}}{{TP}_{k}+{FP}_{k}}\right)$$

(15) $$Recall=\sum_{k=1}^{K}\;\left(\frac{N_{k}}{N}\times \frac{{TP}_{k}}{{TP}_{k}+{FN}_{k}}\right)$$

(16) $$F1=\sum_{k=1}^{K}\;\left(\frac{N_{k}}{N}\times \frac{2\times {Precision}_{k}\times {Recall}_{k}}{{Precision}_{k}+{Recall}_{k}}\right)$$

Here, K denotes the total number of classes, ${TP}_{k}$ denotes the number of samples correctly predicted as class k, N is the total sample count, $N_{k}$ denotes the true sample count of class k, ${FP}_{k}$ denotes samples misclassified as class k but belonging to other classes, and ${FN}_{k}$ denote samples truly belonging to class k but mispredicted as other classes. ${Precision}_{k}$ and ${Recall}_{k}$ denote the precision and recall metrics for class k, respectively.

### 4.5. Experimental Result

**Figure 8:**
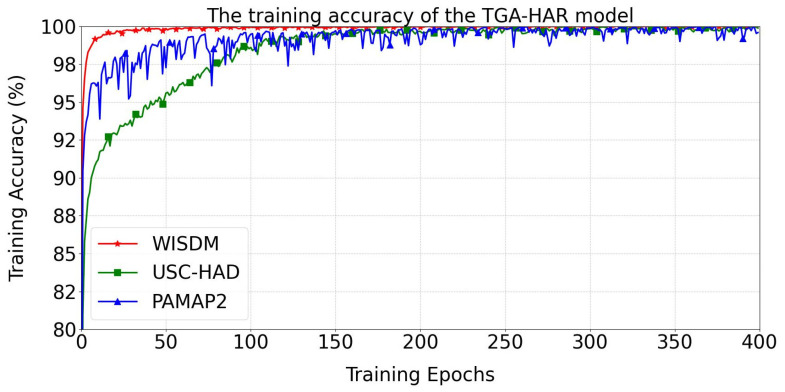
The training accuracy of the TGA-HAR model.

The training accuracy curves of the TGA-HAR model across three human activity datasets (WISDM, USC-HAD, and PAMAP2) are presented in Figure 8. During initial training, the model exhibits rapid accuracy improvement on all datasets. For the WISDM dataset, training accuracy reaches 99% at Epoch 9. The USC-HAD configuration achieves 99% accuracy at Epoch 109, while the PAMAP2 variant attains 99% at Epoch 56. After Epoch 150, both WISDM and USC-HAD configurations stabilize above 99% accuracy. The PAMAP2 implementation exhibits greater volatility.

#### 4.5.1. Overall Performance Evaluation of TGA-HAR on Three Datasets

**Figure 9:**
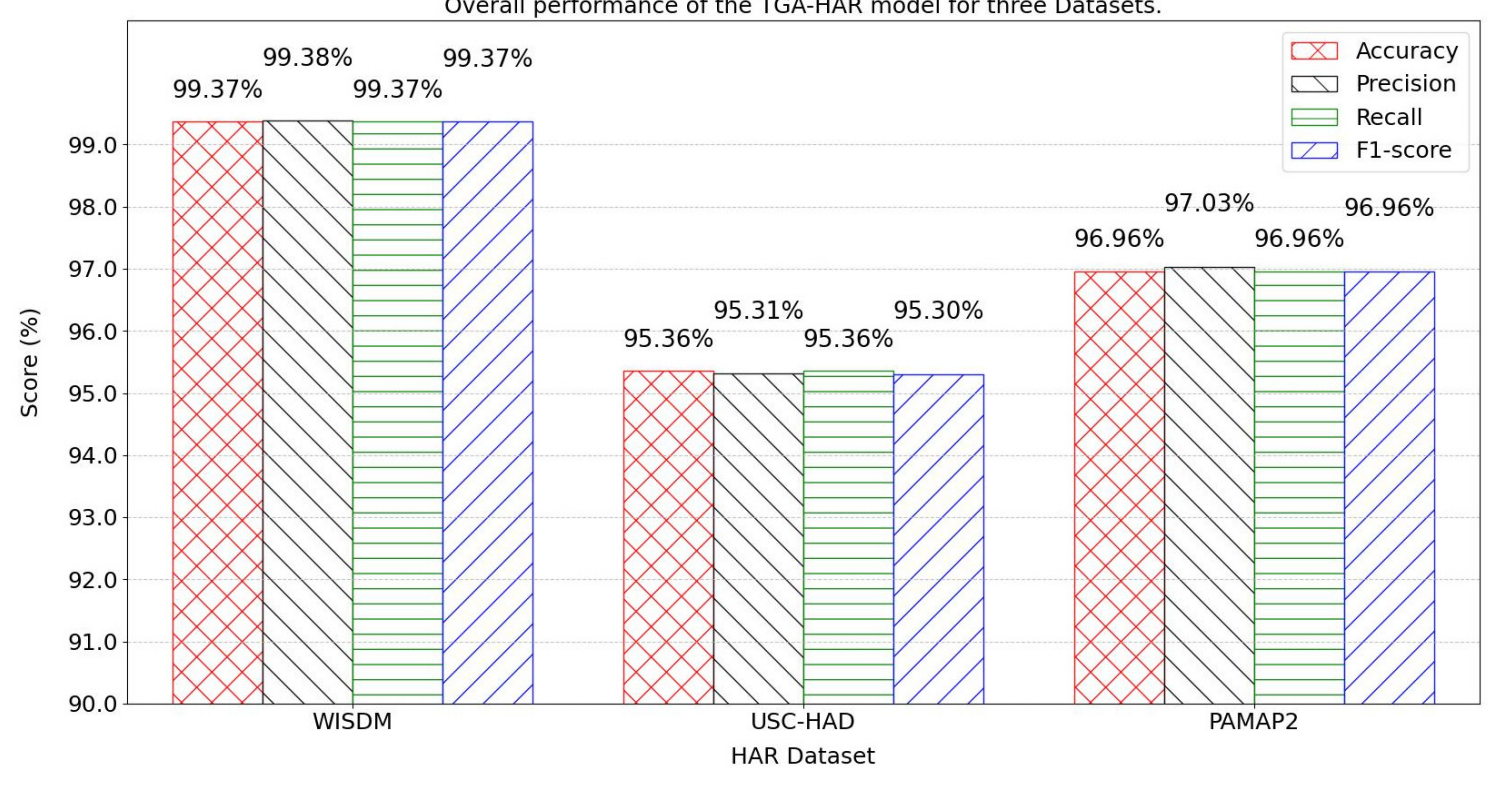
Overall performance of the TGA-HAR model for three Datasets.

Figure 9 illustrates the model’s holistic performance across three datasets.

On the WISDM dataset, the model demonstrates exceptional generalization capability, achieving 99.37% accuracy, 99.38% precision, 99.37% recall, and 99.37% F1 score. This performance may be attributed to standardized activity types and simplified accelerometer-based data collection for basic motions.

For the USC-HAD dataset, experimental evaluation reveals robust recognition performance with 95.36% accuracy, 95.31% precision, 95.36% recall, and a corresponding 95.30% F1-score.

On the PAMAP2 dataset, the model excels in complex scenarios involving multi-sensor fusion, attaining 96.96% accuracy, 96.96% F1 score, 97.03% precision, and 96.96% recall. These metrics confirm that the TGA-HAR model effectively addresses the challenges of fusing heterogeneous sensors (chest-, ankle-, and wrist-mounted monitors) in intricate activity recognition tasks.

#### 4.5.2. Per-Activity Performance Evaluation of TGA-HAR on Three Datasets

**Figure 10:**
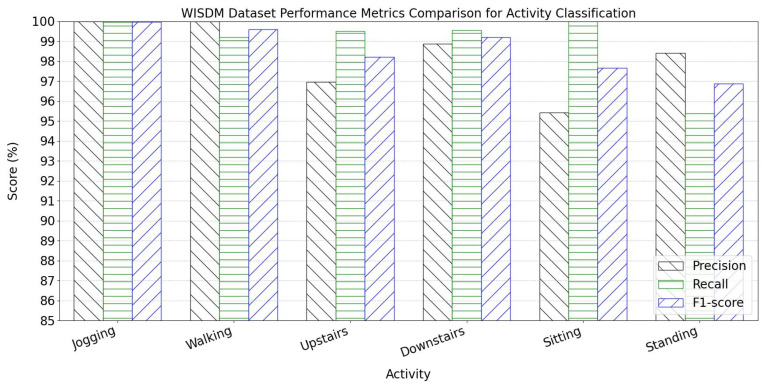
WISDM dataset performance metrics comparison for activity classification.

The per-activity classification metrics for the WISDM dataset are presented in Figure 10. Experiments on six human activities demonstrate outstanding performance across all three core evaluation metrics.

**Figure 11:**
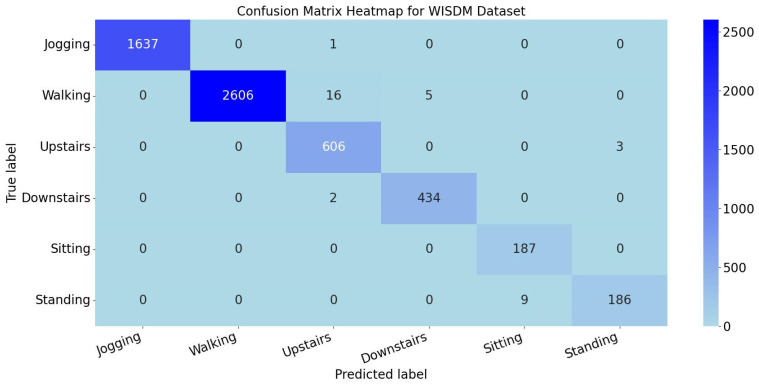
Confusion matrix heatmap for WISDM dataset.

For all six activity classes in WISDM, precision, recall, and F1 score exceed 95%, confirming the model’s stable generalization capability for basic daily activities using triaxial acceleration data. The confusion matrix heatmap is shown in Figure 11.

The heatmap reveals that misclassified cases are minimal across all categories, with an average of 6 misclassifications per activity class. Both the per-activity metrics and heatmap demonstrate the model’s strong discriminative capability in recognizing fundamental motions.

**Figure 12:**
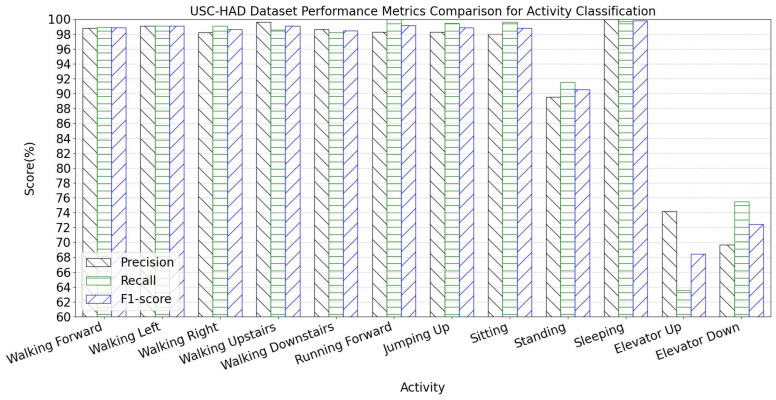
USC-HAD dataset performance metrics comparison for activity classification.

The per-activity classification metrics for the USC-HAD dataset are presented in Figure 12. The TGA-HAR model demonstrates strong classification performance on USC-HAD, indicating high accuracy and robustness in recognizing most activities. However, metrics for elevator-related operations (ascending/descending) are significantly lower, while standing recognition metrics are slightly below other activities. This suggests potential feature similarity challenges between static postures.

**Figure 13:**
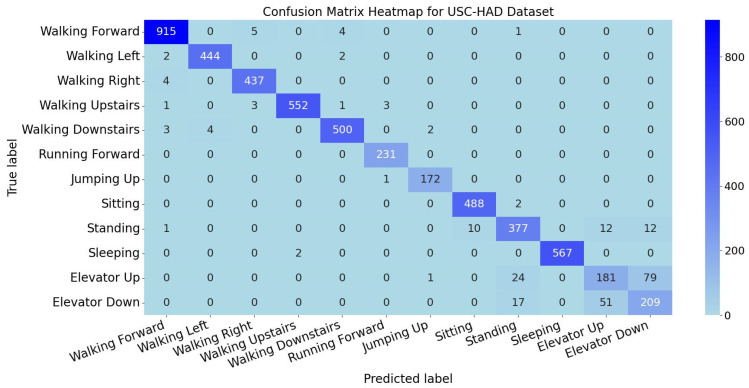
Confusion matrix heatmap for USC-HAD dataset.

The confusion matrix heatmap for activity classification is shown in Figure 13. The heatmap reveals high diagonal accuracy but exhibits localized inter-class confusion. Minor misclassifications in walking-related activities may stem from temporal feature similarities caused by subtle gait variations. Notably, there is pronounced mutual misclassification between elevator ascending/descending and standing categories. Multiple elevator instances are misclassified as standing, and vice versa. This phenomenon occurs because elevator motions exhibit minimal variations in accelerometer and gyroscope signals during steady-state motion (except initial acceleration/final deceleration phases), where sensor readings resemble static standing. For other activities, low off-diagonal values confirm effective class discrimination. Additionally, feature extraction strategies require optimization for elevator and standing scenarios to enhance precision.

**Figure 14:**
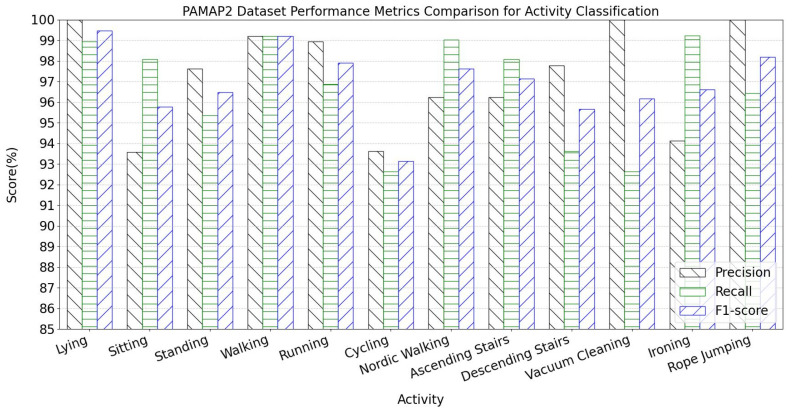
PAMAP2 Dataset Performance Metrics Comparison for Activity Classification.

**Figure 15:**
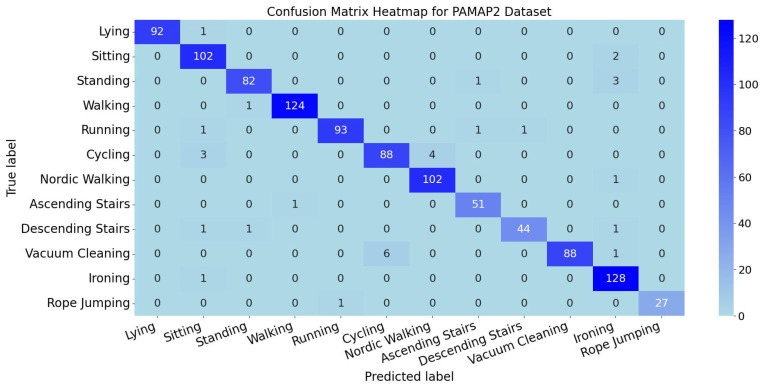
Confusion Matrix Heatmap for PAMAP2 Dataset.

The per-activity classification metrics for the PAMAP2 dataset are shown in Figure 14, while the confusion matrix heatmap for activity classification is displayed in Figure 15. Comprehensive analysis of precision, recall, F1 score, and confusion matrix demonstrates that the TGA-HAR model achieves exceptional classification performance for most activity categories.

Specifically, 12 activity classes exhibit precision, recall, and F1 score consistently maintained above 92%. These values reflect high classification reliability. Confusion matrix analysis further validates the model’s robustness—diagonal elements (correctly classified samples) show significant dominance, with off-diagonal misclassification regions maintaining low values. This confirms the model’s strong discriminative power and generalization capability in multi-class recognition scenarios.

### 4.6. Comparative Experiment

To compare the performance of different models on three datasets, this study designed comparative experiments. The baseline CNN model consists of 6 one-dimensional convolutional layers, each using convolutional kernels of size 5 and 64 channels. After each convolution operation, batch normalization, activation function application, and Dropout regularization are performed sequentially. The RES-CNN model was developed by modifying the aforementioned CNN architecture, where residual connections are introduced after every two convolutional layers to alleviate the vanishing gradient problem in deep networks. For the CNN-LSTM model, a hybrid architecture is adopted. The CNN-LSTM model cascades two layers of unidirectional LSTM networks after the CNN feature extraction module. Each LSTM layer is configured with 64 hidden units to further extract sequential features. The results of the comparative experiments are presented in Table 12.

On the WISDM dataset, the CNN model achieves an accuracy of 96.80% and an F1 score of 96.97%. The Res-CNN model improves these metrics to 96.98% (accuracy) and 97.04% (F1 score). The CNN-LSTM model reaches 98.66% for accuracy and 98.67% for F1 score. The TGA-HAR model (This Study) performs the best, with both accuracy and F1 score at 99.37%. On the USC-HAD dataset, the CNN model yields an accuracy of 88.95% and an F1 score of 89.02%. The Res-CNN model enhances these to 90.73% (accuracy) and 90.66% (F1 score), and the CNN-LSTM model achieves an accuracy of 92.31% with an F1 score of 92.36%. The TGA-HAR model (This Study) leads with an accuracy of 95.36% and an F1 score of 95.30%. For the PAMAP2 dataset, the CNN model obtains an accuracy of 92.21% and an F1 score of 92.24%. The Res-CNN model improves these values to 92.78% (accuracy) and 92.81% (F1 score), respectively, and the CNN-LSTM model further increases them to 94.11% (accuracy) and 94.08% (F1 score). The TGA-HAR model (This Study) again demonstrates the best performance, with both accuracy and F1 score at 96.96%.

In this paper, TGA-HAR adopts a LOSO-like partitioning strategy (i.e., test users are excluded from the training set), while TCN-Attention-HAR uses random 8:2 partitioning of the entire sample set. For cross-dataset performance comparison, TGA-HAR outperforms TCN-Attention-HAR by a slight margin on the WISDM dataset; on the USC-HAD and PAMAP2 datasets, TGA-HAR is slightly inferior to TCN-Attention-HAR, but the performance gap is within 1.5%. This discrepancy stems from the distinct evaluation scenarios—and crucially, TGA-HAR’s architectural design further shapes its adaptability to these scenarios.

Specifically, compared with TCN-Attention-HAR, TGA-HAR incorporates Res-BiGRU, which endows it with advantages in global temporal modeling. The integration of Res-BiGRU enhances TGA-HAR’s capability to capture long-range and bidirectional temporal dependencies, a feature that helps extract common activity patterns across users (rather than relying on individual-specific traits).

In random partitioning, overlap between training and test users allows models to leverage known users’ individual characteristics for recognition—an advantage that TCN-Attention-HAR may exploit more in this scenario. In contrast, LOSO-like partitioning enforces strict user isolation: TGA-HAR is required to extract shared activity features from 80% of users to adapt to unknown users, directly addressing feature shifts caused by individual differences in human activities. This setup evaluates “inter-user generalization ability,” which aligns more with real-world application scenarios but poses higher task difficulty. While TGA-HAR’s Res-BiGRU supports its generalization, the stricter LOSO-like constraint still leads to marginal performance differences on some datasets—a result that reflects the scenario’s inherent complexity rather than a flaw in TGA-HAR’s design. Overall, the TGA-HAR model proposed in this study outperforms the CNN, Res-CNN, and CNN-LSTM models in terms of both accuracy and F1 score across all three datasets. Additionally, the CNN-LSTM model performs better than the Res-CNN and CNN models, while the Res-CNN model only achieves a marginal improvement over the CNN model.

### 4.7. Ablation Experiment

#### 4.7.1. Module Verification

Ablation studies constitute a core methodology for validating the effectiveness of deep learning models. This research employs a hierarchical decoupling strategy to investigate the functional contributions of individual components in temporal pattern recognition.

The TGA-HAR model adopts a multi-stage feature processing architecture:

- Dilated convolutions extract multi-scale local features
- Bidirectional recurrent structures capture temporal dependencies
- Adaptive weighting mechanisms refine the feature space

To verify inter-module synergies, three controlled models are designed:

- GRU-Only: This model retains solely the bidirectional GRU module.
- TCN-Only: This model preserves only the Temporal Convolutional Network (TCN) module.
- TCN-GRU-No-Attention: This model combines TCN and GRU without attention-based feature enhancement.

These controlled models quantify component contributions across feature extraction, temporal propagation, and feature refinement stages by progressively reducing modules. Experiments are conducted on WISDM, USC-HAD, and PAMAP2 datasets under identical hyperparameters and environmental conditions as the full TGA-HAR model, with results detailed in Table 13.

In the relatively simple WISDM scenario, single-module models (TCN-Only or GRU-Only) show certain capabilities in capturing temporal patterns. The TCN-Only model achieves an accuracy of 96.70% and an F1 score of 96.77%. The GRU-Only model attains an accuracy of 96.43% and an F1 score of 96.54%. The TCN-GRU-No-Attention combination, leveraging the complementary nature of local feature extraction by TCN and global temporal feature extraction by GRU, reaches an accuracy of 99.02% and an F1 score of 99.02%. Given the characteristics of the WISDM dataset, the attention mechanism in the subsequent TGA-HAR model further optimizes performance. The TGA-HAR model (This Study) achieves an accuracy of 99.37% and an F1 score of 99.37%, showing a step-by-step improvement compared to the non-attention variant.

In more complex USC-HAD scenarios with intricate activities, standalone models have limitations. The TCN-Only model, when extracting local features, achieves an accuracy of 91.22% and an F1 score of 91.26%. The GRU-Only model, focusing on global temporal features, attains an accuracy of 91.47% and an F1 score of 91.50%. The TCN-GRU-No-Attention combination partially alleviates these problems through cross-scale feature fusion, improving the accuracy by 1.67% (compared to GRU-Only) and the F1 score by 1.62%. Importantly, the attention mechanism in the TGA-HAR model significantly enhances fine—grained activity recognition through weighted feature allocation. It increases the accuracy by 2.22% (compared to TCN-GRU-No-Attention) and the F1 score by 2.18%, finally achieving an accuracy of 95.36% and an F1 score of 95.30%.

For the multimodal complex scenario of PAMAP2, the causal convolutions of the TCN-Only model effectively extract short-term temporal correlations from multi-sensor data, achieving an accuracy of 93.45% and an F1 score of 93.41%. The GRU-Only model captures long-term temporal dependencies from multi-sensor data, attaining an accuracy of 92.88% and an F1 score of 92.89%. The TCN-GRU-No-Attention model, through the joint extraction of features by TCN and GRU, can capture both short-term correlations and long-term dependencies, improving the accuracy by 1.23% (compared to TCN-Only) and the F1 score by 1.28%. The attention layer in the TGA-HAR model dynamically weights the contributions across timesteps, enhancing the classification ability for complex activities. It achieves an accuracy of 96.96% and an F1 score of 96.96%, representing a 2.28% improvement in accuracy compared to the TCN-GRU-No-Attention model.

Performance disparities reveal intrinsic links between component functions and data characteristics. TCN-GRU synergy addresses local-global feature integration challenges, while the attention mechanism enables adaptive optimization for complex scenarios. These elements collectively construct a hierarchical feature processing pipeline for robust activity classification.

#### 4.7.2. The Impact of the Number of GRU Layers on Performance

To investigate the impact of the number of GRU layers on the performance of the TGA-HAR model, this experiment uses the PAMAP2 dataset. The model keeps the convolutional structure of the TCN module, the computational logic of the attention mechanism, and the training hyperparameters exactly consistent. During the experiment, the number of GRU layers in the Res-BiGRU module is sequentially set to 2, 4, and 6. Through this setup, the effect of the layer count on the model is verified. After training is completed, the accuracy and F1 score are evaluated on the test set. The experimental results are shown in Table 14.

When the number of GRU layers is 2, both the model’s accuracy and F1 score reach 96.96%. When the number of layers increases to 4, both metrics rise synchronously to 97.34%, which is an increase of 0.38 percentage points compared to the 2-layer structure. When the number of layers is further increased to 6, the accuracy reaches 97.53%, and this represents an increase of 0.19 percentage points compared to the 4-layer structure. The F1 score in this case is 97.52%, which corresponds to an increase of 0.18 percentage points compared to the 4-layer structure.

From the overall trend, increasing the number of GRU layers gradually enhances the model’s ability to model temporal features. Deeper networks can capture more complex dependency relationships, so the model performance shows a continuous upward trend. However, the magnitude of performance improvement exhibits the characteristic of “diminishing marginal returns”: the performance gain is more significant when increasing from 2 layers to 4 layers, while the magnitude of improvement has narrowed when increasing from 4 layers to 6 layers. Although deeper networks can explore more complex patterns, they are accompanied by a series of issues, such as a sharp increase in the number of parameters, extended computational latency, and greater difficulty in training convergence.

The experimental hardware platform in this study is an NVIDIA GeForce RTX 4050 Laptop GPU with 6 GB of VRAM (Video Random Access Memory). Experimental verification shows that although this platform can support the training and operation of 4-layer and 6-layer GRU (Gated Recurrent Unit) models, it leads to a significant degradation in the model’s recognition speed. Notably, human activity recognition tasks typically require real-time response capabilities (e.g., instant judgment in scenarios involving wearable devices or surveillance systems), and excessively slow recognition speed would undermine the practical application value of the model. After weighing the trade-off between performance and real-time requirements, this study provisionally designates the 2-layer GRU structure as the implementation scheme for the human activity recognition task. If subsequent research can rely on more high-performance hardware platforms (such as high-VRAM GPUs like the RTX 3090/4090, or the construction of multi-GPU parallel architectures), it will be feasible to verify the actual performance of GRU structures with more layers, thereby further tapping the model’s potential in temporal feature modeling.

### 4.8. Evaluation of the TGA-HAR Model in Real-World Environments

Datasets collected in the aforementioned laboratory-controlled environments fail to reproduce issues such as sensor noise, natural interruptions of activities, and variations in device wearing that occur in real-world scenarios. This results in a potential disconnect between the evaluation of model performance and its practical applications.

To address this issue, this subsection adopts the Real-World IDLab Dataset constructed by Stojchevska [51] as the benchmark for evaluation in real-world environments. Characterized by uncontrolled longitudinal data collection, this dataset fully reflects the natural state of human daily activities and thus provides reliable support for the evaluation of model generalization ability.

The Real-World IDLab Dataset employs the Empatica E4 wristband as its data collection device, with a primary focus on utilizing data from the device’s triaxial accelerometer (sampling rate: 32 Hz). Formal data collection involved 18 participants (aged 22–45 years, including 5 females and 13 males), and no researchers were present for observation or monitoring during the collection process. Participants were fully allowed to independently determine activity scenarios (e.g., home, office, outdoor environments) and implementation methods in accordance with their daily routines. This setup naturally captures realistic characteristics such as activity interruptions (e.g., waiting for a red light while walking) and speed variations (e.g., slowing down to avoid pedestrians while cycling)—features that stand in sharp contrast to standardized activity scenarios.

The Real-World IDLab Dataset focuses on 5 categories of core daily activities (Computer Table, Cycling, Running, Standing Still, and Walking). To enhance label quality, it adopts two strategies: model-assisted annotation (pushing activity predictions every 5 min for participants to confirm or correct) and location backtracking (allowing verification of the start and end positions of activity periods). Additionally, targeted cleaning is performed on overlapping labels—for instance, when two mutually exclusive activities are labeled for the same time period, the time boundaries are adjusted or redundant labels are removed. In the data preprocessing stage, the accelerometer signals are scaled to the range of −2 g to +2 g.

Among the dataset we downloaded [52], there is data from 15 participants. We selected the same time window as that in [51], and segmented the data using a 12 s sliding window with a 50% overlap rate, resulting in 3 × 384 matrix samples (3 axial directions × 384 time points). After completing window segmentation, the number of samples for each of the 5 activities per participant is shown in Table 15.

We selected data from a total of 3 participants, which included participant_7, participant_9, and participant_16, as the test set, while the data from the remaining participants was used as the training set. This ensures that the 5 activities have a sufficient number of samples in both the training and test sets. After the division was completed, the samples of the training and test sets are shown in Table 16.

We thus selected the same evaluation metrics as those in [51], namely F1-micro, F1-macro, and Balanced Accuracy. F1-micro, a metric for measuring overall classification accuracy, calculates precision and recall globally by treating all classes as a single whole; F1-macro, which measures the average of classification accuracies across classes, works by first computing precision and recall independently for each class and then averaging the F1 scores of all classes, whereas Balanced Accuracy, a metric that gauges the balance of a classification model’s recall across classes, is defined as the average of the recall rates of all classes. Their calculation formulas are presented in Equations (17)–(19).

(17) $$F1_{micro}=2\;*\;\frac{\left(Precision\;*\;Recall\right)}{\left(Precision+Recall\right)}$$

(18) $$F1_{macro}=\frac{1}{C}\sum_{i=1}^{C}\;2\;*\;\frac{\left(Precision_{i}\;*\;Recall_{i}\right)}{\left(Precision_{i}+Recall_{i}\right)}$$

(19) $$Balanced\;Accuracy=\frac{1}{C}\sum_{i=1}^{C}\;Recall_{i}$$

Here, C denotes the total number of classes. Precision and Recall denote precision and recall at the global level, respectively. $Precision_{i}$ and $Recall_{i}$ denote precision and recall for the i-th class, respectively.

Using the experimental environment and hyperparameter settings consistent with those mentioned above, we completed the model training and then conducted testing using the data from the 4 participants. The results are presented in Table 17.

**Figure 16:**
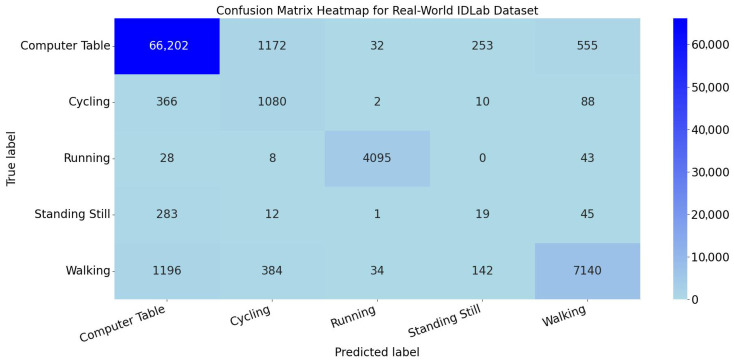
Confusion matrix heatmap for real-world IDLab dataset.

The heatmap of the confusion matrix for the TGA-HAR model on the test set of the Real-World IDLab Dataset is shown in Figure 16. As shown in the confusion matrix heatmap, the TGA-HAR model exhibits distinct classification performance across different activity categories. For Computer Table, Cycling, Running, and Walking, the model correctly identifies most samples (66,202 for Computer Table, 1080 for Cycling, 4095 for Running, and 7140 for Walking). However, for Standing Still, only 19 samples are correctly classified, indicating poor recognition performance.

This performance discrepancy can be attributed to the interplay between the TGA-HAR architecture (TCN-ResBiGRU-Attention) and the inherent characteristics of each activity. Categories like Computer Table, Cycling, Running, and Walking possess distinct and rich temporal features (e.g., stable static patterns for Computer Table, periodic motion rhythms for Cycling and Running). The TCN with dilated convolutions effectively extracts multi-scale temporal features, the Res-BiGRU captures bidirectional temporal dependencies, and the attention mechanism enhances the weights of these discriminative features—thus enabling accurate classification. In contrast, Standing Still has extremely weak temporal dynamics, making its feature distinction from other static categories (e.g., Computer Table) subtle. TCN struggles to extract meaningful patterns from near-static data, Res-BiGRU’s temporal modeling capability is limited in the absence of sequential variation, and the attention mechanism fails to identify prominent feature weights due to insufficient discriminative signals. Additionally, Standing Still has a smaller sample size compared to other categories, leading to inadequate generalization of the model for this class.

While the TGA-HAR model exhibits limited performance in recognizing the “Standing Still” category, it still demonstrates significant advantages based on its TCN-ResBiGRU-Attention architecture—specifically, the dilated convolutions in TCN enable effective extraction of multi-scale temporal features, ResBiGRU robustly captures bidirectional temporal dependencies, and the attention mechanism adaptively enhances the weights of discriminative features. This design allows the model to achieve high accuracy in recognizing dynamic activities (e.g., Cycling, Running, Walking) with rich temporal patterns, as well as static activities like “Computer Table” with distinct motionless characteristics, fully validating its robustness and adaptability in complex real-world HAR tasks.

However, the model’s weakness in recognizing “Standing Still” requires targeted improvements. Data augmentation can be applied to “Standing Still” category data (e.g., adding noise-infused samples or resampling) to expand the training dataset and improve generalization capability; alternatively, the model architecture can be adjusted—such as adding a lightweight static feature modeling branch or optimizing the attention mechanism to prioritize static feature patterns. These measures can improve the recognition performance of “Standing Still” while preserving the model’s advantages in recognizing other categories.

### 4.9. Activity Recognition System

This study aims to achieve complex activity recognition using three wireless IMUs worn by test subjects. The activities correspond to those defined in the PAMAP2 dataset (detailed in Table 7). Sensors are positioned at the dominant wrist, ankle, and chest, with a sampling frequency of 100 Hz.

The recognition system processes 12-dimensional raw data comprising triaxial acceleration and angular velocity from three IMUs. We implement a recognition system to demonstrate experimental outcomes. The system employs the TGA-HAR model, where raw data undergoes preprocessing (Section 4.2) before being fed into the model.

**Figure 17:**
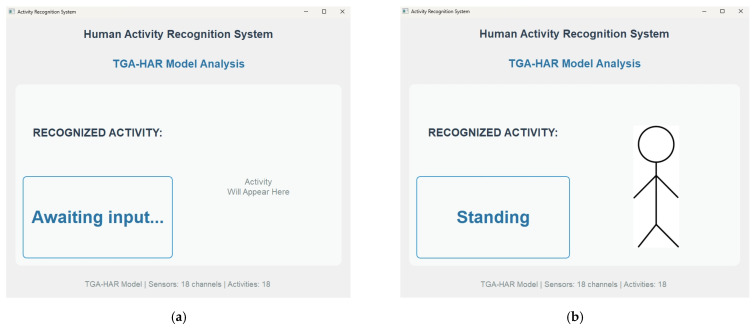
Demonstration of the HAR system using the TGA-HAR model. (**a**) Awaiting input. (**b**) Recognized Activity: Standing.

**Figure A1:**
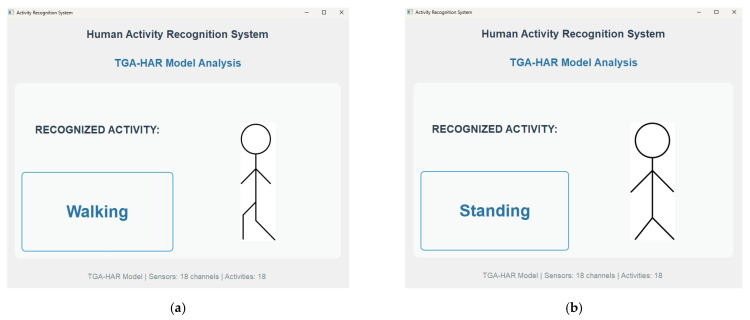

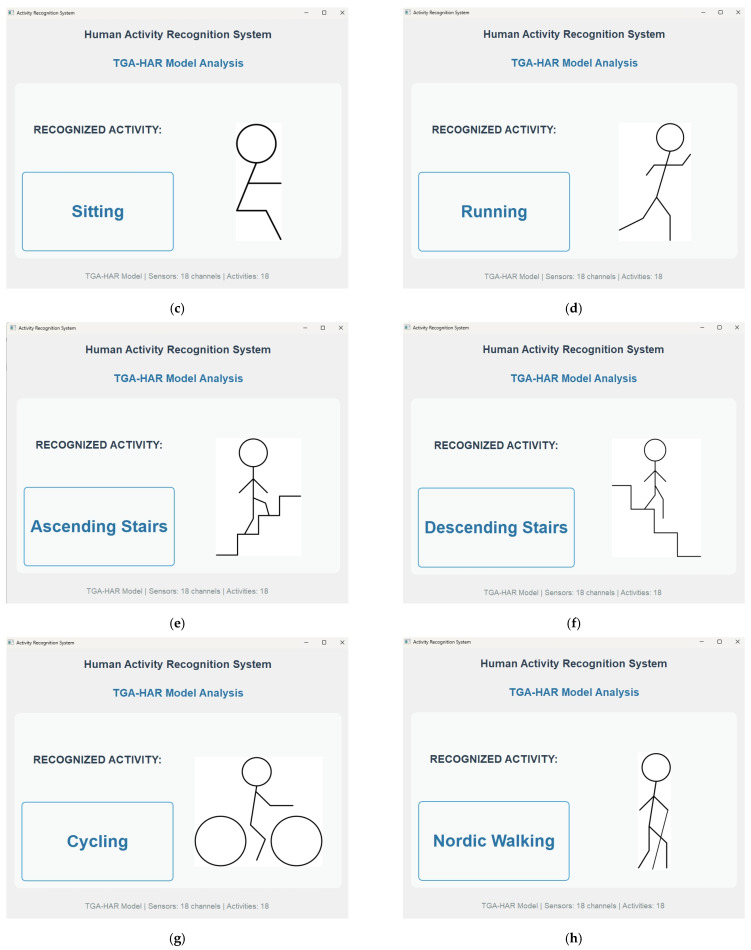

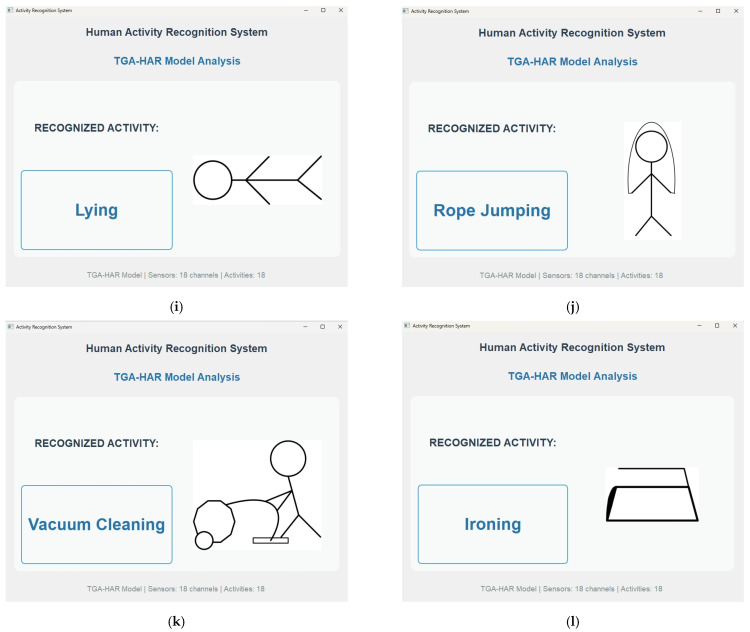
Demonstration of the HAR System Using the TGA-HAR Model (12 Activities). (**a**) Recognized Activity: Walking. (**b**) Recognized Activity: Standing. (**c**) Recognized Activity: Sitting. (**d**) Recognized Activity: Running. (**e**) Recognized Activity: Ascending Stairs. (**f**) Recognized Activity: Descending Stairs. (**g**) Recognized Activity: Cycling. (**h**) Recognized Activity: Nordic Walking. (**i**) Recognized Activity: Lying. (**j**) Recognized Activity: Rope Jumping. (**k**) Recognized Activity: Vacuum Cleaning. (**l**) Recognized Activity: Ironing.

Upon completing activity recognition, the system outputs result as shown in Figure 17. Figure 17a shows standby state awaiting input. Figure 17b show recognized activities during operation. Recognition results for all 18 activities are comprehensively presented in Figure A1.

To scientifically quantify the computational efficiency of the TGA-HAR model, this study utilizes the test set of the PAMAP2 dataset as experimental samples. During experiments, a millisecond-level timer precisely captures the recognition time per sample to evaluate the model’s real-time inference performance. Concurrently, CPU resource utilization is continuously monitored at 1 s intervals throughout the recognition process.

**Figure 18:**
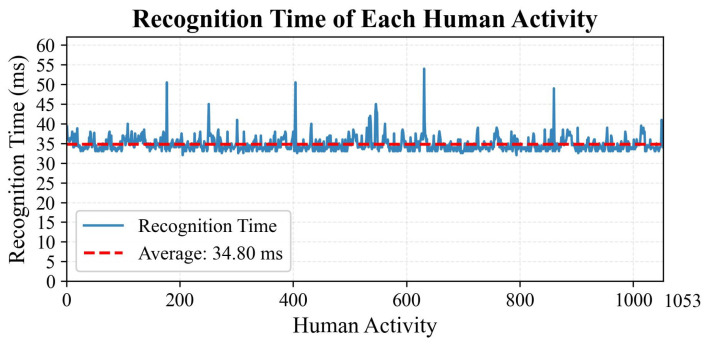
Recognition time of each human activity.

Figure 18 illustrates the recognition time of each human activity of the TGA-HAR model on the PAMAP2 test set. The *x*-axis represents 1053 human activity test samples, while the *y*-axis denotes the inference latency per sample in milliseconds. A dashed horizontal line marks the average inference latency of 34.8 ms, reflecting overall performance. The solid line traces real-time dynamic fluctuations in per-sample latency, demonstrating the model’s behavior during continuous activity recognition. Data distribution reveals that most samples exhibit stable latency fluctuations around the mean, indicating consistent computational efficiency. However, six samples (Nos. 177, 251, 404, 546, 631, 860) show higher latencies (50.51 ms, 45.02 ms, 50.52 ms, 45.00 ms, 53.99 ms, 48.99 ms), attributable to transient resource contention during inference.

**Figure 19:**
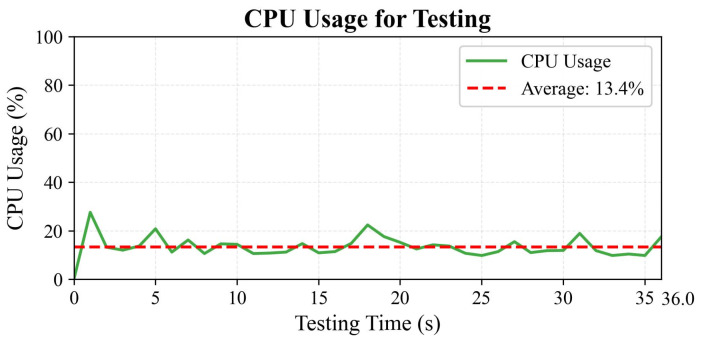
CPU usage for testing.

Figure 19 displays the CPU utilization percentage per second during TGA-HAR model inference across 1053 human activity samples, recorded from 0 to 36.0 s. The *x*-axis represents elapsed time in seconds, while the *y*-axis indicates the CPU usage percentage. A dashed horizontal line marks the average CPU utilization of 13.4% throughout the test. The solid line traces actual second-by-second CPU usage during single-sample inference on the PAMAP2 test set. During the initial 0–2 s, CPU usage peaks at 27.6% due to model initialization and first-frame processing. Subsequent utilization exhibits sawtooth-like fluctuations. As documented in [53], PyTorch employs a decoupled architecture separating control flow (Python) from data flow (C++ operators). Monitoring real-time CPU usage captures switching overhead between these layers, resulting in observed fluctuations—a phenomenon consistent with PyTorch training observations in [54].

## 5. Conclusions

This study proposes TGA-HAR—a three-tier architecture integrating TCN, Res-BiGRU, and an attention mechanism—which achieves significant performance improvements in complex human activity recognition tasks. Experimental results on the WISDM, USC-HAD, and PAMAP2 datasets demonstrate that the model attains accuracies of 99.37%, 95.36%, and 96.96%, with corresponding F1 scores of 99.37%, 95.30%, and 96.96%, respectively. The model employs dilated causal convolutions in the TCN layer for multi-scale local feature extraction, utilizes bidirectional GRU structures to capture long-range temporal dependencies, and enhances classification capability through attention-guided dynamic feature weighting. Tests on real-world datasets show that the TGA-HAR model exhibits good overall recognition capability in HAR tasks. Specifically, it achieves excellent recognition performance for four activity categories, including the dynamic activities Cycling, Running, Walking and the static activity Computer Table, but has limited capability in recognizing Standing Still. Subsequent optimizations can focus on data augmentation for Standing Still, such as adding noise-infused samples or resampling, and adjustments to the model architecture like adding lightweight static feature branches. These efforts aim to improve Standing Still recognition while ensuring the model maintains its strong performance on the four aforementioned categories. During single-sample inference, the TGA-HAR model requires an average latency of 34.80 ms with mean CPU utilization of 13.4%. Ablation studies further confirm synergistic interactions among these modules.

While the TGA-HAR model has shown favorable performance in tests on real-world datasets, further enhancements are still needed to better address practical deployment requirements in the field of HAR. Architecturally, integrating Transformer-based self-attention mechanisms could enhance cross-modal temporal relationship extraction. Furthermore, developing lightweight variants via neural architecture search is crucial for meeting wearable device deployment constraints. While linear interpolation effectively handles short-term sensor signal loss, its adaptability to non-uniform distributions or prolonged missing sequences remains limited. Future work should explore machine learning-based imputation methods to establish more reliable data foundations. This study focuses on the structural design and basic performance verification of the TGA-HAR model, providing a new solution with low-latency characteristics for the field of HAR. However, there is still room for expansion in the systematic demonstration of the “performance–efficiency” trade-off. Future research will supplement horizontal tests on the computational efficiency of mainstream HAR models. By constructing a two-dimensional comparison matrix of “accuracy–inference speed” and “accuracy–resource consumption”, it will examine the TGA-HAR model’s performance in terms of “performance–efficiency” and provide more sufficient data support for the model’s application in practical deployment scenarios such as low-latency embedded devices and lightweight health monitoring terminals. The multi-stage fusion framework proposed in this study offers a novel methodological reference for complex temporal pattern recognition.

## Figures and Tables

**Table 1:** Eight methods in existing HAR research and their used datasets, data acquisition methods, and advantages.

Ref. No.	Dataset	Data Acquisition Method	Method	Advantage
[28]	WISDM, UCI-HAR, PAMAP2	Accelerometer, Gyroscope, IMU	Multi-branch CNN-BiLSTM	Multi-scale Temporal Feature Extraction
[29]	WISDM, UCI-HAR	Accelerometer, Gyroscope	Attention-based Multi-head CNN	SE Module-based Dynamic Channel Weighting
[30]	H-activity, MHEALTH, UCI-HAR	Accelerometer, Gyroscope, Linear acceleration	CNN-LSTM-Self-Attention	Multimodal Temporal Joint Feature Utilization
[31]	PAMAP2, WISDM	Accelerometer, IMU	Spatial Attention CNN + Genetic Algorithm	Wavelet-based Image Encoding and Feature Optimization
[32]	WISDM	Accelerometer	CNN-BiGRU	Bidirectional Gated Units for Long-term Dependencies
[33]	WISDM, PAMAP2, USC-HAD	Accelerometer, Gyroscope, IMU	TCN-Attention-HAR	Knowledge Distillation-based Multi-scale TCN
[34]	MHEALTH and 6 others	Multi sensors	Temporal-Channel Dual-branch Self-Attention Network	Independent Temporal and Channel Dependencies
[35]	KU-HAR	Accelerometer, Gyroscope	Transformer	End-to-End Global Dependencies

**Table 2:** Symbol description table.

**Symbol**	**Description**
K	Kernel size of TCN, K=5
d	Dilation factor of TCN, $d\in \left\{1,\;2,\;4\right\}$
n	Number of convolutional layers within TCN, n∈{1, 2, 3}
t	Time step at moment t
$x_{t-d\times i}^{\left(n\right)}$	Input data at time point t−d×i in the n-th TCN layer
$w_{i}$	Convolution kernel weight at position i
$y_{t}^{\left(n\right)}$	Output of the n-th layer dilated causal convolution
β	Learnable parameter in Swish function (set β=1 in this study)
sigmoid( )	Sigmoid activation function, $sigmoid\left(x\right)=\frac{1}{1+e^{-x}}$
F()	Residual function comprising dilated causal convolution, batch normalization, and activation
$v^{\left(n\right)}$	Input of the n-th residual block or output of the (n−1)-th residual block
$z_{t}$	Update gate state of GRU
$r_{t}$	Reset gate state of GRU
${\tilde{h}}_{t}$	Candidate hidden state of GRU
σ()	Sigmoid activation function,
$W_{z}$, $W_{r}$, W	Trainable weight matrices for GRU update gate, reset gate, and candidate hidden state
$h_{t-1}$	Hidden state corresponding to previous time step t−1
⊙	Element-wise multiplication
$h_{t}$	Final state update of GRU
C	Feature information output by the attention layer

**Table 3:** Summary of data acquisition specifications for the WISDM dataset.

Parameter	Description
Data Acquisition Environment	Laboratory-controlled environment (ensuring data quality)
Number of Participants	36
Number of Activity Types	6
Device Type	Smartphone accelerometer
Number of Devices Worn	1
Wearing Position	In their front pants leg pocket
Transmission Mode	File transfer during post-processing
Sampling Frequency	20 Hz
Sampling Duration	Approximately 15.25 h

**Table 4:** Summary of data acquisition specifications for the USC-HAD dataset.

Parameter	Description
Data Acquisition Environment	Laboratory-controlled environment (wired connection ensures signal quality)
Number of Participants	14
Number of Activity Types	12
Device Type	IMU
Number of Devices Worn	1
Wearing Position	At their front right hip
Transmission Mode	Wired connection
Sampling Frequency	100 Hz
Sampling Duration	Average 6 h per participant

**Table 5:** Description of 12 activity types in the USC-HAD dataset.

No.	Activity	Description
1	Walking Forward	The subject walks forward in a straight line.
2	Walking Left	The subject walks counter-clockwise in a full circle.
3	Walking Right	The subject walks clockwise in a full circle
4	Walking Upstairs	The subject goes up multiple flights.
5	Walking Downstairs	The subject goes down multiple fights.
6	Running Forward	The subject runs forward in a straight line.
7	Jumping	The subject stays at the same position and continuously jumps up and down.
8	Sitting	The subject sits on a chair either working or resting. Fidgeting is also considered to belong to this class.
9	Standing	The subject stands and talks to someone.
10	Sleeping	The subject sleeps or lies down on a bed.
11	Elevator Up	The subject rides in an ascending elevator.
12	Elevator Down	The subject rides in a descending elevator.

**Table 6:** Summary of Data Acquisition Specifications for the PAMAP2 Dataset.

Parameter	Description
Data Acquisition Environment	Daily life environment (partial signal loss during acquisition)
Number of Participants	9
Number of Activity Types	18
Device Type	IMU, Heart rate monitor
Number of Devices Worn	IMU × 3, Heart rate monitor × 1
Wearing Position	Three IMUs: wrist (dominant side), chest, ankle (dominant side); Heart rate monitor: chest
Transmission Mode	Wireless transmission
Sampling Frequency	100 Hz
Sampling Duration	Over 10 h per participant

**Table 7:** Description of 18 Activity Types in the PAMAP2 Dataset.

No.	Activity	Description
1	Lying	Lying quietly while doing nothing, small movements—e.g., changing the lying posture—are allowed.
2	Sitting	Sitting in a chair in whatever posture the subject feels comfortable, changing sitting postures is also allowed.
3	Standing	Consists of standing still or standing still and talking, possibly gesticulating.
4	Ironing	Ironing 1–2 shirts or T-shirts.
5	Vacuuming	Vacuum cleaning one or two office rooms (which includes moving objects, e.g., chairs, placed on the floor).
6	Ascending stairs	Ascending stairs was performed in a building between the ground and the top floors, a distance of five floors had to be covered going upstairs.
7	Descending stairs	Descending stairs was performed in a building between the ground and the top floors, a distance of five floors had to be covered going downstairs.
8	Normal walking	Walking outside with moderate to brisk pace with a speed of 4–6 km/h, according to what was suitable for the subject.
9	Nordic walking	Nordic walking was performed outside on asphaltic terrain, using asphalt pads on the walking poles (it has to be noted, that none of the subjects was very familiar with this sport activity).
10	Cycling	Cycling was performed outside with a real bike with slow to moderate pace, as if the subject would bike to work or bike for pleasure (but not as a sport activity).
11	Running	Running meant jogging outside with a suitable speed for the individual subjects.
12	Rope jumping	The subjects used the technique most suitable for them, which mainly consisted of the basic jump (where both feet jump at the same time over the rope) or the alternate foot jump (where alternate feet are used to jump off the ground).
13	Watching TV	Watching TV at home, in whatever posture—lying, siting—the subject feels comfortable.
14	Computer work	Working normally in the office.
15	Car driving	Driving between office and subject’s home.
16	Folding laundry	Folding shirts, T-shirts and/or bed linens.
17	House cleaning	Dusting some shelves, including removing books and other things and putting them back again onto the shelves.
18	Playing soccer	Playing 1 vs. 1 or 2 vs. 1, running with the ball, dribbling, passing the ball and shooting the ball on goal.

**Table 8:** Training–test set sample distribution for human activity recognition datasets.

Dataset	Post-Preprocessing Samples	Training Set Samples	Test Set Samples
WISDM	26,447	20,838	5609
USC-HAD	20,769	15,449	5320
PAMAP2	7494	6441	1053

**Table 9:** Experimental environment.

Type	Specification
CPU	Intel Core i7-13700H
GPU	NVIDlA GeForce RTX 4050 Laptop GPU (6 GB)
Memory	16 GB DDR5 4800 MHz
Python	3.11
PyTorch	2.2.0
CUDA	11.8

**Table 10:** The hyperparameter configuration.

Hyperparameter	Description
Optimizer	Adam
Epochs	400
Batch Size	300
Learning Rate	0.001
Loss Function	Cross Entropy

**Table 11:** TGA-HAR model parameters.

Model Block		Set	
TCN	TCN Block 1	*K*	5
		*d*	1
		Filters	64
		Dropout rate	0.2
	TCN Block 2	*K*	5
		*d*	2
		Filters	64
		Dropout rate	0.2
	TCN Block 3	*K*	5
		*d*	4
		Filters	64
		Dropout rate	0.2
Res-BiGRU	BiGRU 1	Neuron	64
	BiGRU 2	Neuron	64
Attention Layer			
Fully Connected Layer (FC Layer)	FC Layer 1	Neuron	64
	FC Layer 2	Neuron	32
	FC Layer 3	Neuron	Activity number

**Table 12:** Comparative experiment results.

Model	Dataset					
	WISDM		USC-HAD		PAMAP2	
	Accuracy (%)	F1-Score (%)	Accuracy (%)	F1-Score (%)	Accuracy (%)	F1-Score (%)
CNN	96.80	96.97	88.95	89.02	92.21	92.24
Res-CNN	96.98	97.04	90.73	90.66	92.78	92.81
CNN-LSTM	98.66	98.67	92.31	92.36	94.11	94.08
TCN-Attention-HAR [34]	99.03	98.50	96.32	94.23	98.35	98.37
TGA-HAR (This Study)	99.37	99.37	95.36	95.30	96.96	96.96

**Table 13:** Ablation experiment results.

Model	Dataset					
	WISDM		USC-HAD		PAMAP2	
	Accuracy (%)	F1-Score (%)	Accuracy (%)	F1-Score (%)	Accuracy (%)	F1-Score (%)
TCN-Only	96.70	96.77	91.22	91.26	93.45	93.41
GRU-Only	96.43	96.54	91.47	91.50	92.88	92.89
TCN-GRU-No-Attention	99.02	99.02	93.14	93.12	94.68	94.69
TGA-HAR (This Study)	99.37	99.37	95.36	95.30	96.96	96.96

**Table 14:** Comparison of accuracy and F1-Score of the TGA-HAR model on the PAMAP2 dataset under different numbers of GRU Layers.

The Number of GRU Layers	Accuracy (%)	F1-Score (%)
2	96.96	96.96
4	97.34	97.34
6	97.53	97.52

**Table 15:** Sample Distribution of the Real-World IDLab Dataset After Window Segmentation.

Participant_ID	Computer Table	Cycling	Running	Standing Still	Walking
Participant_0	29,970	138	0	187	1111
Participant_3	34,725	1576	29	0	8726
Participant_5	46,789	397	0	48	2228
Participant_6	34,155	0	0	1208	3581
Participant_7	23,705	1250	0	129	4567
Participant_8	7621	595	0	0	104
Participant_9	1428	296	4174	0	2563
Participant_10	230	470	767	0	288
Participant_11	774	416	0	219	3693
Participant_12	564	0	395	0	388
Participant_13	6294	295	1305	0	1901
Participant_14	16,487	3173	7146	645	10,351
Participant_15	260	20	196	0	184
Participant_16	43,081	0	0	231	1766
Participant_17	16,019	444	0	47	1117
Sum	262,102	9070	14,012	2714	42,568

**Table 16:** Sample Distribution of Training Set and Test Set for the Real-World IDLab Dataset.

Dataset Setup	Computer Table	Cycling	Running	Standing Still	Walking	Sum
Training Set	193,888	7524	9838	2354	33,672	247,276
Test Set	68,214	1546	4174	360	8896	83,190

**Table 17:** Experimental results for the real-world IDLab dataset.

Metrics	Value (%)
$F1_{micro}$	94.41
$F1_{macro}$	67.36
Balanced Accuracy	70.11

## Data Availability

The datasets analyzed during the current study are openly available in the following repositories: the WISDM Activity Recognition Dataset in the WISDM Lab repository at [http://www.cis.fordham.edu/wisdm/dataset.php, accessed on 24 March 2025]; the USC-HAD Human Activity Dataset in the USC-SIPI repository at [https://sipi.usc.edu/HAD/, accessed on 24 March 2025]; the PAMAP2 Physical Activity Monitoring Dataset in the UCI Machine Learning Repository at [https://archive.ics.uci.edu/dataset/231/pamap2+physical+activity+monitoring, accessed on 24 March 2025]; and the Real-World IDLab Dataset in the IDLab Cloud repository at [https://cloud.ilabt.imec.be/index.php/s/2zkXxEDTTQgkN5y, accessed on 22 August 2025].
